# Risk prediction in patients with classical low-flow, low-gradient aortic stenosis undergoing surgical intervention

**DOI:** 10.3389/fcvm.2023.1197408

**Published:** 2023-06-12

**Authors:** Fernanda Castiglioni Tessari, Maria Antonieta Albanez A. de M. Lopes, Carlos M. Campos, Vitor Emer Egypto Rosa, Roney Orismar Sampaio, Frederico José Mendes Mendonça Soares, Rener Romulo Souza Lopes, Daniella Cian Nazzetta, Fábio Sândoli de Brito Jr, Henrique Barbosa Ribeiro, Marcelo L. C. Vieira, Wilson Mathias, Joao Ricardo Cordeiro Fernandes, Mariana Pezzute Lopes, Carlos E. Rochitte, Pablo M. A. Pomerantzeff, Alexandre Abizaid, Flavio Tarasoutchi

**Affiliations:** ^1^ Instituto do Coracao (InCor), Hospital das Clinicas HCFMUSP, Faculdade de Medicina, Universidade de Sao Paulo, Sao Paulo, Brazil; ^2^ Department of Hemodynamic, Real Hospital Português, Recife, Brazil; ^3^ Department of Hemodynamic, Instituto Prevent Senior, Sao Paulo, Brazil

**Keywords:** aortic stenosis, risk prediction, valve surgery, echocardiography, cardiac magnetic resonance

## Abstract

**Introduction:**

Classical low-flow, low-gradient aortic stenosis (LFLG-AS) is an advanced stage of aortic stenosis, which has a poor prognosis with medical treatment and a high operative mortality after surgical aortic valve replacement (SAVR). There is currently a paucity of information regarding the current prognosis of classical LFLG-AS patients undergoing SAVR and the lack of a reliable risk assessment tool for this particular subset of AS patients. The present study aims to assess mortality predictors in a population of classical LFLG-AS patients undergoing SAVR.

**Methods:**

This is a prospective study including 41 consecutive classical LFLG-AS patients (aortic valve area ≤1.0 cm^2^, mean transaortic gradient <40 mmHg, left ventricular ejection fraction <50%). All patients underwent dobutamine stress echocardiography (DSE), 3D echocardiography, and T1 mapping cardiac magnetic resonance (CMR). Patients with pseudo-severe aortic stenosis were excluded. Patients were divided into groups according to the median value of the mean transaortic gradient (≤25 and >25 mmHg). All-cause, intraprocedural, 30-day, and 1-year mortality rates were evaluated.

**Results:**

All of the patients had degenerative aortic stenosis, with a median age of 66 (60–73) years; most of the patients were men (83%). The median EuroSCORE II was 2.19% (1.5%–4.78%), and the median STS was 2.19% (1.6%–3.99%). On DSE, 73.2% had flow reserve (FR), i.e., an increase in stroke volume ≥20% during DSE, with no significant differences between groups. On CMR, late gadolinium enhancement mass was lower in the group with mean transaortic gradient >25 mmHg [2.0 (0.0–8.9) g vs. 8.5 (2.3–15.0) g; *p* = 0.034), and myocardium extracellular volume (ECV) and indexed ECV were similar between groups. The 30-day and 1-year mortality rates were 14.6% and 43.8%, respectively. The median follow-up was 4.1 (0.3–5.1) years. By multivariate analysis adjusted for FR, only the mean transaortic gradient was an independent predictor of mortality (hazard ratio: 0.923, 95% confidence interval: 0.864–0.986, *p* = 0.019). A mean transaortic gradient ≤25 mmHg was associated with higher all-cause mortality rates (log-rank *p* = 0.038), while there was no difference in mortality regarding FR status (log-rank *p* = 0.114).

**Conclusions:**

In patients with classical LFLG-AS undergoing SAVR, the mean transaortic gradient was the only independent mortality predictor in patients with LFLG-AS, especially if ≤25 mmHg. The absence of left ventricular FR had no prognostic impact on long-term outcomes.

## Introduction

Classical low-flow and low-gradient aortic stenosis (LFLG-AS) is a challenging clinical entity that has garnered increased recognition in recent years. It is characterized by a mismatch between a reduced aortic valve area (AVA) and a nonsevere transaortic mean gradient in patients with reduced left ventricular ejection fraction (LVEF). Recent studies report that classical LFLG-AS accounts for 5%–10% of patients with severe aortic stenosis (AS) (1, 2).

Although aortic valve replacement (AVR) is a well-established management strategy for classical LFLG-AS, studies on interventional risk prediction are largely noncontemporary and have primarily focused on transcatheter AVR (TAVR) (3–8). For instance, once considered a survival marker, left ventricular flow reserve (FR) has recently come under scrutiny for its prognostic relevance (2, 3, 7). Furthermore, earlier studies have examined a heterogeneous population of low-gradient AS, and their findings may not be entirely generalizable to classical LFLG-AS patients (5, 6, 9).

Therefore, there is currently a paucity of information regarding the current prognosis of classical LFLG-AS patients undergoing surgical AVR (SAVR) and the lack of a reliable risk assessment tool for this particular subset of AS patients. The present study aims to assess mortality predictors in a population of classical LFLG-AS patients undergoing SAVR.

## Methods

### Study population and protocol

**Figure 1:**
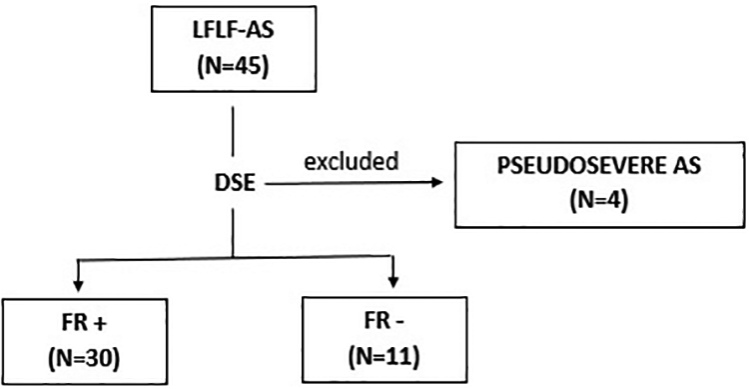
Study flowchart. Selection of the study population. All patients without flow reserve on dobutamine stress echocardiography underwent an aortic valve calcium score on computed tomography. AS, aortic stenosis; DSE, dobutamine stress echocardiography; FR, flow reserve; LFLG, low-flow, low-gradient.

This study included a prospective cohort comprising 41 consecutive patients with classical LFLG-AS (i.e., AVA ≤1.0 cm^2^, mean transaortic gradient <40 mmHg, and LVEF <50%) and SAVR indication. Exclusion criteria were (I) severe primary mitral or aortic regurgitation, (II) moderate-to-severe mitral stenosis, (III) cardiac magnetic resonance (CMR)-incompatible devices or contraindications to gadolinium-enhanced CMR, (IV) previous valve surgery, (V) nonischemic cardiomyopathies, and/or (VI) diagnosis of pseudo-severe AS on dobutamine stress echocardiography (DSE) (*n* = 4) (Figure 1). A dedicated electronic case report form was designed to collect baseline characteristics, procedure details, and clinical follow-up data. All patients underwent DSE, 2D and 3D transthoracic echocardiography, CMR with T1 mapping and late gadolinium enhancement (LGE) evaluation, and laboratory examination. Coronary angiography was performed in each patient, and coronary artery disease was considered in the presence of >50% luminal stenosis on the major epicardial coronary artery.

Patients were divided into groups according to the mean transaortic gradient ≤25 or >25 mmHg. In order to obtain two groups with a balanced number of patients, this cutoff was determined from the median value of the mean transaortic gradient. All-cause mortality, intraprocedural mortality, 30-day mortality, 1-year mortality, stroke, myocardial infarction, pacemaker implantation, pericardial effusion, postprocedural atrial fibrillation, and reintervention were evaluated. Written informed consent was provided from all the patients, and the study protocol was reviewed and approved by the local institutional ethics committee.

### Echocardiography

All transthoracic echocardiographs were analyzed in a central echocardiography laboratory. DSE was performed as previously described (2, 10) using a commercially available ultrasound system (Vivid 9; GE Healthcare, Milwaukee, WI, United States), as rest 2D echocardiography. The presence of FR was defined as an increase in stroke volume ≥20% during DSE. True-severe AS was defined by the presence of a mean transaortic gradient ≥40 mmHg with an AVA ≤1.0 cm^2^ during DSE, and pseudo-severe AS was defined by a mean transaortic gradient <40 mmHg and an AVA >1.0 cm^2^. In the absence of FR, AS severity was confirmed by the computed tomography aortic valve calcium score and considered severe if ≥1,300 AU in women and ≥2,000 AU in men (11, 12). Echocardiographic parameters were measured using the methods recommended by the American Society of Echocardiography (13). Left ventricular global longitudinal strain was measured by speckle tracking with dedicated commercial software (EchoPAC V 110.0.x; GE Healthcare, Milwaukee, WI, United States), as previously reported (14). Three-dimensional echocardiography was performed using a commercially available ultrasound system (EPIQ Ultrasound, with a 5 MHz transducer; Philips, Andover, MA, United States), and the parameters were analyzed according to standard recommendations (15).

### CMR protocol

All CMR exams were performed using a clinical 1.5-T CMR scanner (Achieva; Philips, Best, the Netherlands), and the analyses were performed by two experienced investigators in a central CMR core laboratory at our institution. The analyses were performed using CVi42 (Circle CVi; Calgary, Canada) software, and images were acquired and coupled to the electrocardiograph during breath-hold. LGE imaging for myocardial fibrosis was performed 10 min after a bolus (0.2 mmol/kg body weight) of gadolinium-based contrast. Native T1 mapping and T1 postcontrast mapping were calculated before and 15–20 min after the intravenous injection of 0.2 mmol/kg gadolinium-based contrast, respectively, using the modified look-locker inversion-recovery sequence, performed in expiratory apnea, into three segments of the left ventricle short axis (base, mid, and apex). The T1 value was calculated as a global myocardial T1 (pre- and postgadolinium) value and excluded subendocardial and transmural fibrosis areas (segments with mid-wall LGE were included). Atrial fibrillation patients had controlled heart rates (60–90 bpm) during the exam, and T1 mapping image acquisition was repeated, taking into account the average of T1 values in both pre- and postgadolinium sequences. The extracellular volume (ECV) was calculated using the following formula: ECV_myo _= (1 − hematocrit) × ΔR1_myo_/ΔR1_blood_, where ΔR1 = (1/T1 precontrast − 1/T1 postcontrast) (16). To calculate the indexed ECV (iECV), the following formula was used: ECV (excluding areas of focal fibrosis) × indexed left ventricular end-diastolic myocardial volume (17).

### Data analysis

Continuous variables were presented as median (25th–75th percentile). Categorical variables were presented as percentages. The Mann–Whitney *U*-test was applied for continuous variables, and the Fisher exact test or *χ*^2^ test was applied for categorical variables, as appropriate. Cox regression analysis was used to evaluate the predictors of all-cause mortality. Variables with a *p* < 0.05 in univariate analyses were entered in the multivariable model and adjusted for FR. Survival curves were estimated using the Kaplan–Meier method and compared between patient groups with the log-rank test. All tests were two-tailed, and a *p* > 0.05 was used to indicate statistical significance. All analyses were conducted using statistical package SPSS, version 20 (IBM, Armonk, NY, United States).

## Results

### Patient characteristics

Clinical characteristics and laboratory data are summarized in Table 1. A total of 41 patients with severe degenerative LFLG-AS were enrolled. The median age was 66 (60.0–73.5) years, with a male predominance (82.9%). Functional class III or IV by NYHA classification was present in 51.2%, 26.8% had angina, and only 3% had syncope. There was a high prevalence of comorbidities such as hypertension (68.3%), diabetes (39%), chronic kidney disease (39%), and atrial fibrillation (26.8%), and 36.6% had concomitant coronary artery disease. Almost one-third of the patients had left bundle branch block (29.3%) on the baseline electrocardiogram. The median EuroSCORE II was 2.19% (1.5%–4.78%), and the median STS was 2.19% (1.6%–3.99%). Patients were compared regarding the two-dimensional echocardiographic mean transaortic gradient. Twenty patients had a mean transaortic gradient ≤25 mmHg, and 21 patients had a mean transaortic gradient >25 mmHg. There were no differences in clinical and laboratory data regarding group definition (Table 1).

**Table 1:** Baseline clinical and laboratory data of the study population.

Variable	Total (*n* = 41)	Mean transaortic gradient ≤25 (*n* = 20)	Mean transaortic gradient >25 (*n* = 21)	*p*-value
**Clinical data**				
Age, years	66 (60.0–73.5)	69 (61.7–73.7)	65 (57.5–73.5)	0.290
Body surface area, m^2^	1.80 (1.71–1.92)	1.79 (1.72–1.92)	1.81 (1.67–1.95)	0.917
Male sex	34 (82.9)	18 (90.0)	16 (76.2)	0.410
Diabetes	16 (39.0)	9 (45.0)	7 (33.3)	0.656
Hypertension	28 (68.3)	14 (70.0)	14 (66.7)	1.000
Atrial fibrillation	11 (26.8)	7 (35.0)	4 (19.0)	0.424
Coronary artery disease	15 (36.6)	10 (50.0)	5 (23.8)	0.157
One vessel	3 (7.3)	1 (5.0)	2 (9.5)	
Two vessels	5 (12.2)	2 (9.5)	3 (15.0)	
Three vessels	7 (17.1)	1 (4.8)	6 (30.0)	
Previous CABG	6 (14.6)	4 (20.0)	2 (9.5)	0.410
EuroSCORE II, %	2.19 (1.50–4.78)	3.32 (1.72–5.25)	1.79 (1.13–3.90)	0.101
STS, %	2.19 (1.60–3.99)	3.14 (1.68–3.83)	1.90 (1.40–4.44)	0.351
**Symptoms**				
NYHA III/IV	21 (51.2)	11 (55.0)	10 (47.6)	0.873
Angina	11 (26.8)	6 (28.6)	5 (25.0)	1.000
Syncope	3 (7.3)	1 (5.0)	2 (9.5)	1.000
**Medications**				
ACE inhibitors or ARB	29 (70.7)	14 (70.0)	15 (71.4)	1.000
Βeta blockers	21 (51.2)	12 (60.0)	9 (42.9)	0.432
Antiplatelets	23 (56.1)	12 (60.0)	11 (52.4)	0.860
Diuretics	35 (85.4)	15 (75.0)	20 (95.2)	0.093
Statins	29 (70.7)	12 (60.0)	17 (81.0)	0.258
Digoxin	9 (22.0)	5 (25.0)	4 (19.0)	0.719
Oral anticoagulation	11 (26.8)	7 (35.0)	4 (19.0)	0.424
**ECG**				
Left bundle branch block	12 (29.3)	6 (30.0)	6 (28.6)	1.000
Right bundle branch block	2 (4.9)	–	2 (9.5)	0.488
**Laboratory data**				
Hemoglobin, mg/dl	13.5 (12.7–14.3)	13.3 (12.5–14.3)	13.7 (12.7–14.6)	0.309
Hematocrit, %	41 (39–44)	40 (38–43)	41 (39–45)	0.160
eGFR, ml/min	55 (46–64)	48 (36–61)	59 (45–72)	0.130
CKD (eGFR < 60 ml/min)	16 (39.0)	11 (55.0)	5 (23.8)	0.084
Troponin I, ng/ml	0.043 (0.025–0.102)	0.043 (0.020–0.102)	0.045 (0.026–0.105)	0.758
B-type natriuretic peptide, pg/ml	378 (138–659)	259 (138–630)	469 (131–710)	0.739
C-reactive protein, mg/dl	2.9 (1.5–6.8)	2.6 (1.5–6.1)	3.4 (1.5–8.0)	0.771

ACE, angiotensin-converting enzyme; ARB, angiotensin receptor blocker; CABG, coronary artery bypass graft; CKD, chronic kidney disease; ECG, electrocardiogram; eGFR, estimated glomerular filtration rate; NYHA, New York Heart Association.

Values are median (25th–75th percentile) or *n* (%).

Bold values denote statistical significance.

### Echocardiography data

Baseline transthoracic and DSE data are summarized in Table 2. There were no differences between the groups regarding two- and three-dimensional echocardiography in terms of morphological and functional characteristics, except that patients in the mean transaortic gradient >25 mmHg group had, as expected, a higher mean transaortic gradient [33 (30–36) vs. 21 (19–23) mmHg; *p* < 0.001], peak transaortic gradient [53 (49–61) vs. 36 (30–39) mmHg; *p* < 0.001], and peak aortic valve velocity [3.64 (3.5–3.9) vs. 2.99 (2.70–3.11) m/s; *p* < 0.001]. The median stroke volume index was 34 (30–40) ml/m^2^, the global longitudinal strain was 10% (8.7%–12%) [–], and the valvuloarterial impedance was 5.2 (4.7–5.7) mmHg/ml/m^2^, with no difference between groups. Regarding three-dimensional echocardiography, data between groups were also similar, with a median LVEF of 31 (24–39)%, AVA of 0.83 (0.66–0.90) cm^2^, and AVA index of 0.43 (0.37–0.47) cm^2^/m^2^.

**Table 2:** Baseline two- and three-dimensional echocardiography and dobutamine stress echocardiography data.

Variable	Total (*n* = 41)	Mean transaortic gradient ≤25 (*n* = 20)	Mean transaortic gradient >25 (*n* = 21)	*p*-value
**Baseline 2D echocardiography**				
LVEF, %	35 (28–43)	34 (26–41)	38 (28–43)	0.461
LVEDD, mm	58 (55–63)	57 (53–64)	58 (55–63)	0.824
LVESD, mm	46 (40–52)	47 (38–52)	46 (40–52)	0.989
LVEDV, ml	190 (171–243)	184 (156–243)	207 (178–245)	0.289
LVESV, ml	128 (102–163)	135 (102–169)	124 (102–163)	0.968
LV mass, g/m^2^	142 (128–170)	138 (129–160)	146 (119–182)	0.321
Mean transaortic gradient, mmHg	26 (21–33)	21 (19–23)	33 (30–36)	**<0**.**001**
Peak transaortic gradient, mmHg	41 (36–53)	36 (30–39)	53 (49–61)	**<0**.**001**
Peak aortic valve velocity, m/s	3.2 (2.99–3.64)	2.99 (2.70–3.11)	3.64 (3.5–3.9)	**<0**.**001**
PASP, mmHg	43 (34–50)	44 (32–51)	43 (35–50)	0.799
Aortic diameter, cm	33 (30–35.75)	33 (30–36.75)	32 (30–35)	0.989
Left atrium diameter, cm	48 (42.5–50)	48 (43.25–49.75)	46 (41–51)	0.927
Septum, cm	11 (9–13)	11.5 (9–13)	11 (9.5–12.5)	0.906
Posterior wall, cm	10 (9.5–12.0)	10.5 (9.0–11.7)	10 (10–12)	0.661
Aortic valve area, cm^2^	0.85 (0.66–0.95)	0.88 (0.80–0.95)	0.82 (0.62–0.96)	0.758
Aortic valve area index, cm^2^/m^2^	0.47 (0.36–0.51)	0.47 (0.38–0.52)	0.46 (0.34–0.50)	0.383
Stroke volume index, ml/m^2^	34 (30–40)	35 (31–42)	33 (30–40)	0.901
Valvuloarterial impedance, mmHg/ml/m^2^	5.2 (4.7–5.7)	5.0 (4.6–5.6)	5.3 (4.8–5.8)	0.512
Global longitudinal strain ([–] %)	10 (8.7–12)	10 (9–12)	10 (6.8–12)	0.620
Moderate/severe functional mitral regurgitation	13 (31.7)	6 (30.0)	7 (33.3)	1.000
Moderate/severe functional tricuspid regurgitation	5 (12.2)	3 (15.0)	2 (9.5)	0.663
Segmental dysfunction	9 (22.0)	5 (25.0)	4 (19.0)	0.719
Diastolic dysfunction				0.502
Grade 1	8 (29.6)	5 (33.3)	3 (25.0)	
Grade 2	11 (40.7)	6 (40.0)	5 (41.7)	
Grade 3	4 (14.8)	3 (20.0)	1 (8.3)	
**Baseline 3D echocardiography**				
LVEF, %	31 (24–39)	31 (24–38)	35 (24–39)	0.718
LVEDV, ml	173 (150–212)	159 (148–206)	185 (166–218)	0.183
LVESV, ml	115 (87–145)	111 (84–138)	123 (90–155)	0.445
Aortic valve area, cm^2^	0.83 (0.66–0.90)	0.85 (0.70–0.91)	0.70 (0.61–0.90)	0.327
Aortic valve area index, cm^2^/m^2^	0.43 (0.37–0.47)	0.46 (0.41–0.49)	0.41 (0.36–0.45)	0.134
**Dobutamine stress echocardiography**				
Flow reserve	30 (73.2)	14 (70.0)	16 (76.2)	0.925
Basal aortic valve area, cm^2^	0.80 (0.72–0.96)	0.84 (0.69–0.98)	0.80 (0.73–0.95)	1.000
Peak stress aortic valve area, cm^2^	0.85 (0.70–0.97)	0.89 (0.63–1.00)	0.80 (0.71–0.90)	0.443
Basal mean transaortic gradient, mmHg	29 (22–32)	22 (18–30)	31 (27–34)	**0**.**002**
Peak stress mean transaortic gradient, mmHg	35 (29–47)	32 (22–45)	42 (33–49)	**0**.**030**
Basal stroke volume index, ml/m^2^	29.7 (24.6–37.7)	25.5 (20.2–31.2)	32.2 (27.0–45.7)	**0**.**012**
Peak stress stroke volume index, ml/m^2^	36.5 (29.4–42.0)	30.6 (28.0–38.7)	39 (35–45)	0.063
Basal indexed flow rate, ml/m^2^ s	101 (85–126)	94 (73–121)	118 (88–145)	0.190
Peak indexed flow rate, ml/m^2^ s	137 (106–162)	106 (95–139)	143 (126–164)	0.037

DSE, dobutamine stress echocardiography; Gm, mean transaortic gradient; LV, left ventricular; LVEDD, left ventricular end-diastolic diameter; LVESD, left ventricular end-systolic diameter; LVEDV, left ventricular end-diastolic volume; LVEF, left ventricular ejection fraction; LVESV, left ventricular end-systolic volume; PASP, pulmonary arterial systolic pressure.

Values are median (25th–75th percentile) or *n* (%).

Bold values denote statistical significance.

On DSE, FR was present in most of the patients (73.2%), with no significant differences between the groups. Peak stress parameters did not differ significantly between the groups, except for the peak stress mean transaortic gradient, which was higher in the mean transaortic gradient ≤25 mmHg group [42 (33–49) vs. 32 (22–45) mmHg; *p* = 0.030]. In contrast to the 2D echocardiography evaluation, the median stroke volume index was higher in the mean transaortic gradient >25 mmHg group [32.2 (27.0–45.7) vs. 25.5 (20.2–31.2) ml/m^2^, *p* = 0.012]. However, this difference was no longer observed after dobutamine infusion [39 (35–45) vs. 30.6 (28.0–38.7) ml/m^2^, *p* = 0.063].

### CMR data

CMR data are shown in Table 3 and were similar between the groups, except for LGE mass, which was lower in the mean transaortic gradient >25 mmHg group [2.0 (0.0–8.9) vs. 8.5 (2.3–15.0) g; *p* = 0.034]. Delayed-enhancement images showed a transmural pattern in 29.3% and a mesocardial pattern in 26.8%. Interstitial fibrosis analyses were also similar between groups: overall ECVs including and excluding positive delayed-enhancement were 28.9% (26.8%–33.2%) and 28.7% (26.3%–31.9%), respectively, and iECV was 34.9 (24.9–40.8) ml/m^2^.

**Table 3:** Cardiac magnetic resonance data.

Variable	Total (*n* = 41)	Mean transaortic gradient ≤25 (*n* = 20)	Mean transaortic gradient >25 (*n* = 21)	*p*-value
RVEDV index, ml/m^2^	60.2 (54.3–85.5)	63.5 (52.3–90.5)	59.4 (56.5–75.0)	0.629
RVESV index, ml/m^2^	32.4 (20.5–44.6)	31 (18.7–49.2)	32.4 (22.3–41.9)	0.764
RV ejection fraction, %	47 (30–63)	56 (30–66)	45 (31–58)	0.206
LVEDV index, ml/m^2^	115 (87–137)	111 (87–138)	120 (87–137)	0.958
LVESV index, ml/m^2^	78 (56–98)	79 (49–101)	78 (57–98)	0.979
LVEF, %	32 (25–43)	34 (23–46)	31 (28–43)	0.865
Aortic valve area, cm^2^	0.8 (0.6–0.9)	0.8 (0.7–0.9)	0.7 (0.6–0.9)	0.235
Peak transaortic gradient, mmHg	36 (28–63)	33 (25–50)	40 (34–81)	0.134
Mean transaortic gradient, mmHg	9 (5–13)	8 (5–11)	11 (6–17)	0.174
Positive mesocardial delayed-enhancement images	11 (26.8)	6 (30)	5 (23.8)	0.925
Positive transmural delayed-enhancement images	12 (29.3)	7 (35.0)	5 (23.8)	0.657
LV mass, g	199 (168–247)	200 (151–255)	199 (174–231)	0.927
LGE mass, g	4.9 (0.0–12.7)	8.5 (2.3–15.0)	2.0 (0.0–8.9)	0.034
ECV including positive delayed-enhancement images, %	28.9 (26.8–33.2)	29.6 (26.9–33.8)	28.7 (26.5–32.0)	0.341
ECV excluding positive delayed-enhancement images, %	28.7 (26.3–31.9)	28.9 (26.7–33.0)	27.1 (25.8–30.0)	0.291
iECV, ml/m^2^	34.9 (24.9–40.8)	37.1 (26.9–41.5)	34.1 (24.8–38.7)	0.404

ECV, extracellular volume; Gm, mean transaortic gradient; iECV, indexed extracellular volume; LGE, late gadolinium enhancement; LV, left ventricular; LVEDV, left ventricular end-diastolic volume; LVEF, left ventricular ejection fraction; LVESV, left ventricular end-systolic volume; RV, right ventricular; RVEDV, right ventricular end-diastolic volume; RVESV, right ventricular end-systolic volume.

Values are median (25th–75th percentile) or *n* (%).

### Procedural data and outcomes

Procedural data and postprocedural outcomes are summarized in Table 4. The occurrence of postprocedural complications was evaluated and compared between the groups, with no statistical difference. Infection was the most frequent complication, followed by atrial fibrillation (43.9% and 19.5%, respectively). A definitive pacemaker was implanted in three (7.3%) patients; stroke and pericardial effusion both occurred in only one (2.4%) patient. Concomitant coronary artery bypass graft was performed in three patients from each group, with no statistical difference between groups. There was no ascending aortic procedure nor mitral valve intervention. Cardiopulmonary bypass time was the only variable with a difference between the groups and was lower in the patients with mean transaortic gradient >25 mmHg [60 (52–73) vs. 77 (60–100) min; *p* = 0.023]. Both 30-day and 1-year mortality rates were also similar, and there was no intraprocedural mortality.

**Table 4:** Procedure data and post-procedure outcomes.

Variable	Total (*n* = 41)	Mean transaortic gradient ≤25 (*n* = 20)	Mean transaortic gradient >25 (*n* = 21)	*p*-value
Cardiopulmonary bypass time, min	69 (55–92)	77 (60–100)	60 (52–73)	**0**.**023**
Cross-clamp time, min	51 (40–68)	60 (42–83)	44 (39–60)	0.099
Concomitant CABG	6 (14.6)	3 (15.0)	3 (14.3)	1.000
30-day mortality	6 (14.6)	2 (10.0)	4 (19.0)	0.663
1-year mortality	14 (43.8)	9 (45.0)	5 (41.7)	1.000
Stroke	1 (2.4)	1 (5.0)	—	0.488
Definitive pacemaker	3 (7.3)	1 (5.0)	2 (9.5)	1.000
Pericardial effusion	1 (2.4)	—	1 (4.8)	1.000
Infection	18 (43.9)	10 (50.0)	8 (38.1)	0.536
Atrial fibrillation	8 (19.5)	5 (25.0)	3 (14.3)	0.454
Reintervention	2 (4.9)	—	2 (9.5)	0.488

CABG, coronary artery bypass graft.

Values are median (25th–75th percentile) or *n* (%).

Bold values denote statistical significance.

**Figure 2:**
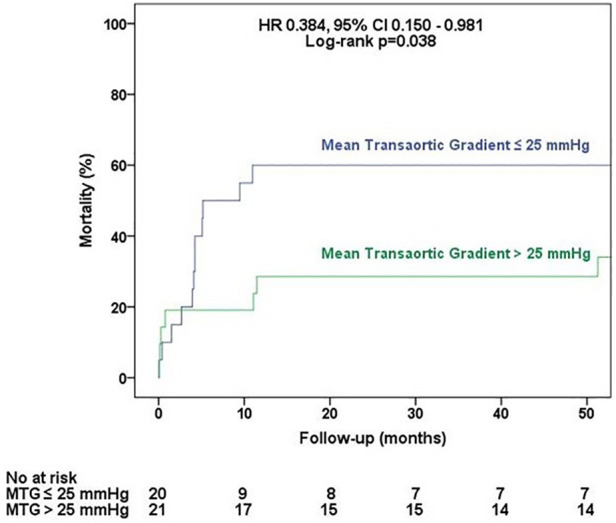
All-cause mortality according to the mean transaortic gradient. Survival curves according to the mean transaortic gradient ≤25 and >25 mmHg at rest echocardiography.

**Figure 3:**
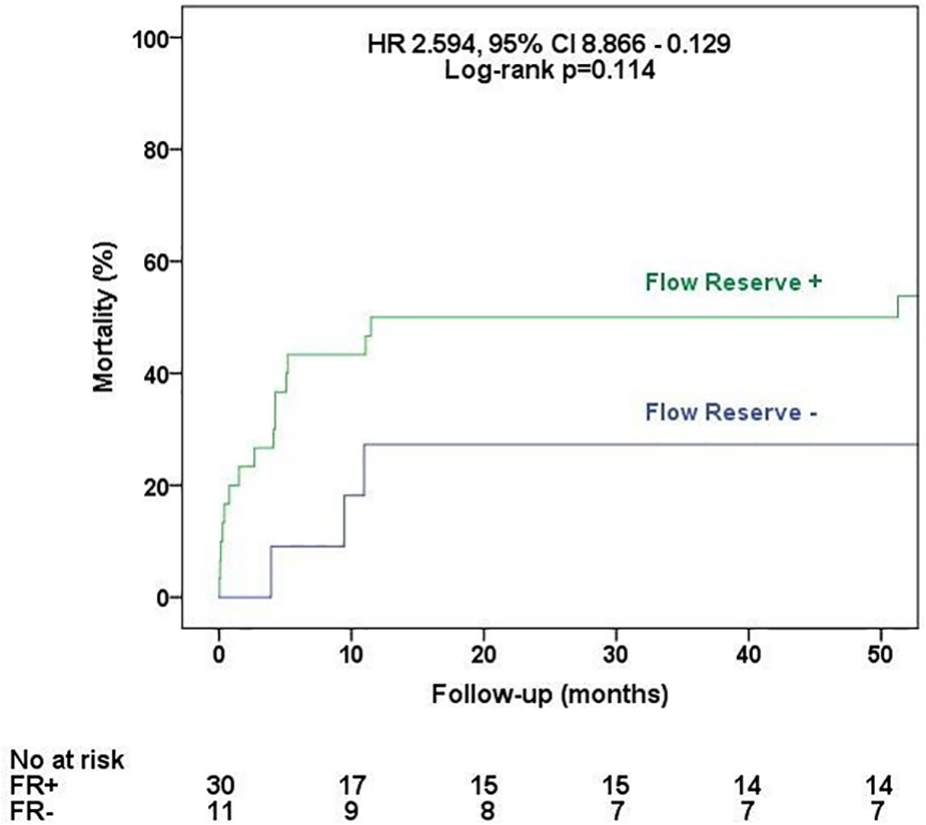
All-cause mortality according to the flow reserve status. Survival curves according to the presence or absence of flow reserve at dobutamine stress echocardiography.

All-cause mortality was evaluated with a median follow-up of 4.1 (0.3–5.1) years. In the univariate analysis of predictors of all-cause mortality (Table 5 and Supplementary Table S1), three variables were associated with the outcome: STS [hazard ratio (HR): 1.253, 95% confidence interval (CI): 1.019–1.541, *p* = 0.032], 2D echocardiographic mean transaortic gradient (HR: 0.932, 95% CI: 0.882–0.984, *p* = 0.011), and C-reactive protein (HR: 1.033, 95% CI: 1.008–1.059, *p* = 0.011). However, in the multivariate analysis adjusted for FR, only 2D echocardiographic mean transaortic gradient was an independent predictor of mortality (HR: 0.908, 95% CI: 0.837–0.984, *p* = 0.019). As demonstrated in Figure 2, patients with transaortic mean gradient >25 mmHg had a lower rate of all-cause mortality during the follow-up (log-rank *p* = 0.038), while the presence of FR (Figure 3) had no impact on mortality (log-rank *p* = 0.239).

**Table 5:** Univariate and multivariate analyses of predictors of all-cause mortality adjusted for flow reserve.

	HR	Univariate analysis		*p-*value	HR	Multivariate analysis		*p*-value
		95.0% CI				95.0% CI		
		Lower limit	Upper limit			Lower limit	Upper limit	
STS, %	1.253	1.019	1.541	0.032	1.157	0.927	1.444	0.197
2D echocardiographic mean transaortic gradient, mmHg	0.932	0.882	0.984	0.011	0.908	0.837	0.984	0.019
C-reactive protein, mg/dl	1.033	1.008	1.059	0.011	1.026	1.000	1.053	0.050
Flow reserve	2.594	0.759	8.866	0.129	3.103	0.728	13.217	0.126

CI, confidence interval; HR, hazard ratio.

## Discussion

The main findings of the present study, including patients with classical LFLG-AS undergoing SAVR, can be summarized as follows: (1) the rest transaortic mean gradient was the only independent predictor of mortality; and (2) the absence of left ventricular FR was not associated with worse outcomes in a median of 4-year follow-up.

Classical LFLG-AS affects only 5%–10% of the population with AS and represents an advanced stage of the disease, as the impaired left ventricle is unable to generate a rest high transaortic gradient. This entity is related to poor clinical outcomes, and conservative treatment has been associated with mortality rates as high as 60% in 2 years (8, 18). However, despite an increased risk for adverse outcomes even with surgical or transcatheter AVR, robust data show that aortic intervention is still beneficial compared to the traditional approach (9, 19–21). Thus, it is imperative to recognize the patients with classical LFLG-AS who will benefit from SAVR, and studies on this topic are scarce.

The absence of FR on DSE has been described for a long time as a predictor of higher mortality in patients undergoing SAVR, with an operative mortality rate of about 30% vs. 5%–7% in the presence of FR (8). However, several recent studies have tried to refute such a theory. First, a prospective study including patients with classical LFLG-AS evaluated by CMR demonstrated that the absence of FR is not related to the amount of diffuse interstitial fibrosis assessed by ECV and iECV, refuting the previous idea that patients without FR could have larger amounts of fibrosis and therefore an increased operative risk (2). Second, the TOPAS-TAVI registry demonstrated that the absence of FR was neither associated with higher mortality rates nor with lower LVEF recovery after TAVR (7). This is in line with Buchanan et al. (3), who showed that FR did not predict all-cause mortality at 30 days or 1 year after TAVR, and Sato et al. (20), who also demonstrated that FR was not a predictor of better survival (3, 20). However, Sato et al. (20) were the only ones who evaluated SAVR patients, but still, the analysis did not differentiate those who underwent TAVR procedures (20). Thus, the results of these studies may not necessarily extend to patients undergoing SAVR exclusively, as those included in the present study.

The multicentric TOPAS registry evaluated predictors of poor outcomes in patients with low-gradient AS undergoing SAVR, TAVR, or a medical approach (5, 7, 9). The TOPAS-TAVI registry demonstrated that lower hemoglobin levels, chronic obstructive pulmonary disease, and moderate-to-severe residual aortic regurgitation were predictors of poor outcomes in a 2-year follow-up after TAVR (7). Another substudy demonstrated a prognostic value of both brain natriuretic peptide (BNP) and high-sensitivity troponin T levels in patients with classical and paradoxical LFLG-AS. Moreover, when occurring simultaneously, values ≥550 pg/ml and ≥15 ng/L, respectively, were independent predictors of 2-year mortality, with higher mortality compared to the elevation of none or only one biomarker (5). A third study on patients undergoing CMR demonstrated that impaired ventricular global longitudinal strain (<−11%), higher ECV (>28%) and LGE presence were predictors of worse outcomes, with a cumulative effect on survival analysis curves (9). However, in these last two studies, the pooled data included not only classical LFLG-AS but also paradoxical AS and normal-flow low-gradient AS (5, 9). It is important to note that AS subtypes have different pathophysiologies since classical LFLG-AS is similar to heart failure with reduced ejection fraction, while the paradoxical AS has similar characteristics to heart failure with preserved ejection fraction. Thus, it is possible that mortality predictors may be different for such pathologies, and they should be studied separately.

Studies including only classical LFLG-AS patients undergoing SAVR are scarce and noncontemporary, revealing high surgical mortality but even worse outcomes with conservative medical treatment (4, 6, 8, 10, 18). Such data corroborate the indication of intervention in patients with classical LFLG-AS and the need for new risk prediction strategies. In line with previous studies, we demonstrated that a lower transaortic mean gradient was associated with worse outcomes, especially if ≤25 mmHg (6, 8). It is noteworthy that, despite there being no difference in LVEF between groups, these patients with lower gradients also presented lower cardiac output at rest and a trend to lower cardiac output at stress, as demonstrated by the basal and peak stroke volume index on DSE, which could indicate a more advanced stage of the disease and, hence, a poor prognosis.

The median value of the mean transaortic gradient (≤25 mmHg) was arbitrarily chosen as the cutoff to divide the population into two groups to obtain two groups with a comparable number of patients. Baseline characteristics were similar between them, except for LGE mass, which was higher among patients with a mean transaortic gradient ≤25 mmHg. Interestingly, different from the present study, LGE was also one of the mortality predictors described by Fukui et al. (9), and this discrepancy could be explained by the higher LGE prevalence demonstrated by that study (67% vs. 53.7% in the present study). In addition, the different populations included in their study (i.e., paradoxical LFLG and normal-flow low-gradient AS, besides classical LFLG-AS) may also impact the results (9). Patients with a mean transaortic gradient of ≤25 mmHg had longer cardiopulmonary bypass time. However, no surgical technical issues could account for this observation, as the rates of coronary artery bypass graft procedures were similar between the groups, and patients did not undergo any other concomitant interventions. Moreover, although longer cardiopulmonary bypass time may influence prognosis and introduce potential bias in the present study, it was not deemed significant as a predictor of mortality in the analysis.

Due to its less invasive nature, TAVR appears to have a higher survival benefit than SAVR (19). The present study demonstrated that classical LFLG-AS patients undergoing SAVR had a higher 30-day mortality rate (14.6%) than that predicted by EuroSCORE II [2.19% (1.50%–4.78%)] and the STS score [2.19% (1.60%–3.99%)]. Meanwhile, the TOPAS registry demonstrated a different scenario in those patients undergoing TAVR, with a 30-day mortality rate of 3.8%, which was lower than the mortality risk predicted by the STS score and EuroSCORE II [7.7% (5.3%–12.0%) and 10.5% (5.5%–17.3%), respectively] (7). However, currently available surgical risk scores may not adequately assess the operative risk, and further studies are needed to obtain better prediction tools for this specific high-risk population.

### Study limitations

This is a single-center study with a heterogeneous population and a relatively small number of patients, although large for this entity. The small number of events may have impacted the mortality prediction, despite being enough to fit the developed model (22). In this cohort, different from the former studies, only patients with classical LFLG-AS undergoing SAVR were included. Moreover, further randomized studies are needed to compare treatment strategies in classical LGLF-AS patients (TAVR vs. SAVR).

## Conclusion

In patients with classical LFLG-AS undergoing SAVR, the echocardiographic rest transaortic mean gradient was the only independent predictor of mortality. In addition, the absence of left ventricular FR was not associated with worse outcomes, confirming the diagnostic rather than the prognostic value of FR.

## Data Availability

The original contributions presented in the study are included in the article/Supplementary Material, further inquiries can be directed to the corresponding author.
