# Supplementary material for: Risk prediction in patients with classical low-flow, low-gradient aortic stenosis undergoing surgical intervention
Source: Front Cardiovasc Med. 2023 Jun 12;10:1197408. doi: 10.3389/fcvm.2023.1197408 (PMC10291604; doi:10.3389/fcvm.2023.1197408)
Supplement: Supplementary file 1 [file Table1.docx]

**Supplemental Table 1. Univariated analysis of predictors of all-cause mortality**

|  | **HR** | **95.0% CI** | | ***P* value** | |  |
| --- | --- | --- | --- | --- | --- | --- |
|  |  | **Lower limit** | **Upper limit** | |  | |
| **Clinical data** |  |  |  | |  | |
| Age, years | 1.020 | 0.962 | 1.541 | | 0.511 | |
| Body surface area, m^2^ | 2.054 | 0.107 | 39.574 | | 0.633 | |
| Female sex | 1.210 | 0.404 | 3.628 | | 0.734 | |
| Diabetes | 1.321 | 0.546 | 3.193 | | 0.537 | |
| Hypertension | 3.135 | 0.915 | 10.737 | | 0.069 | |
| Atrial fibrillation | 1.723 | 0.686 | 4.332 | | 0.247 | |
| Coronary artery disease  One vessel  Two vessels  Three vessels | 1.938  0.000  2.125  3.726 | 0.794  0.000  0.588  1.312 | 4.728  7.676  10.581 | | 0.146  0.982  0.250  0.014 | |
| Previous CABG | 1.101 | 0.322 | 3.766 | | 0.878 | |
| EuroSCORE II, % | 1.054 | 0.907 | 1.223 | | 0.494 | |
| STS, % | 1.253 | 1.019 | 1.541 | | **0.032** | |
| **Symptoms** |  |  |  | |  | |
| NYHA III/IV | 0.787 | 0.325 | 1.903 | | 0.595 | |
| Angina | 0.627 | 0.209 | 1.876 | | 0.403 | |
| Syncope | 0.043 | 0.000 | 31.360 | | 0.349 | |
| **ECG** |  |  |  | |  | |
| Left bundle branch block | 1.255 | 0.500 | 3.151 | | 0.629 | |
| Right bundle branch block | 0.044 | 0.000 | 90.108 | | 0.422 | |
| **Laboratory data** |  |  |  | |  | |
| Hemoglobin, mg/dl | 0.793 | 0.571 | 1.101 | | 0.166 | |
| Hematocrit, % | 0.910 | 0.814 | 1.018 | | 0.101 | |
| eGFR, mL/min | 0.990 | 0.968 | 1.014 | | 0.411 | |
| CKD (eGFR < 60 mL/min) |  |  |  | |  | |
| Troponin I, ng/mL | 6.532 | 0.419 | 101.902 | | 0.181 | |
| B-type natriuretic peptide, pg/mL | 1.001 | 1.000 | 1.001 | | 0.133 | |
| C-reactive protein, mg/dL | 1.033 | 1.008 | 1.059 | | **0.011** | |
| **Baseline 2D echocardiography** |  |  |  | |  | |
| LVEF, % | 0.987 | 0.936 | 1.040 | | 0.617 | |
| LVEDD, mm | 0.997 | 0.935 | 1.063 | | 0.920 | |
| LVESD, mm | 1.014 | 0.956 | 1.075 | | 0.648 | |
| LVEDV, mL | 0.997 | 0.987 | 1.006 | | 0.469 | |
| LVESV, mL | 1.000 | 0.989 | 1.011 | | 0.996 | |
| LV mass, g/m^2^ | 0.995 | 0.984 | 1.005 | | 0.327 | |
| Mean transaortic gradient, mmHg | 0.932 | 0.882 | 0.984 | | **0.011** | |
| PASP, mmHg | 1.008 | 0.963 | 1.055 | | 0.733 | |
| Septum, cm | 1.038 | 0.862 | 1.249 | | 0.695 | |
| Posterior wall, cm | 1.012 | 0.784 | 1.305 | | 0.930 | |
| Aortic valve area, cm^2^ | 1.623 | 0.124 | 21.300 | | 0.712 | |
| Aortic valve area index, cm^2^/m^2^ | 1.292 | 0.009 | 176.471 | | 0.919 | |
| Stroke volume index, mL/m² | 1.003 | 0.951 | 1.057 | | 0.916 | |
| Valvuloarterial impedance, mmHg/mL/m^2^ | 0.775 | 0.480 | 1.251 | | 0.296 | |
| Global longitudinal strain ([−] %) | 1.033 | 0.894 | 1.195 | | 0.657 | |
| Moderate/severe functional mitral regurgitation | 0.702 | 0.255 | 1.934 | | 0.494 | |
| Moderate/severe functional tricuspid regurgitation | 2.594 | 0.855 | 7.869 | | 0.092 | |
| Diastolic dysfunction  Grade 1  Grade 2  Grade 3 | 0.819  0.988  0.866 | 0.149  0.199  0.121 | 4.505  4.911  6.168 | | 0.819  0.989  0.885 | |
| **Baseline 3D echocardiography** |  |  |  | |  | |
| LVEF, % | 0.986 | 0.936 | 1.039 | | 0.603 | |
| LVEDV, mL | 0.995 | 0.985 | 1.006 | | 0.374 | |
| LVESV, mL | 0.999 | 0.987 | 1.011 | | 0.863 | |
| Aortic valve area, cm^2^ | 2.202 | 0.131 | 37.046 | | 0.584 | |
| Aortic valve area index, cm^2^/m^2^ | 1.845 | 0.008 | 447.236 | | 0.827 | |
| **Dobutamine stress echocardiography** |  |  |  | |  | |
| Flow reserve | 2.594 | 0.759 | 8.866 | | 0.129 | |
| Basal aortic valve area, cm^2^ | 0.147 | 0.010 | 2.176 | | 0.163 | |
| Peak stress aortic valve area, cm^2^ | 0.548 | 0.035 | 8.657 | | 0.669 | |
| Basal mean transaortic gradient, mmHg | 0.955 | 0.902 | 1.012 | | 0.117 | |
| Peak stress mean transaortic gradient, mmHg | 1.005 | 0.970 | 1.041 | | 0.778 | |
| Basal stroke volume index, mL/m^2^ | 1.008 | 0.964 | 1.054 | | 0.735 | |
| Peak stress stroke volume index, mL/m^2^ | 0.999 | 0.935 | 1.069 | | 0.988 | |
| Basal indexed flow rate, mL/m² ·s | 1.001 | 0.983 | 1.020 | | 0.893 | |
| Peak indexed flow rate, mL/m² ·s | 1.007 | 0.990 | 1.025 | | 0.407 | |
| **Cardiac Magnetic Resonance** |  |  |  | |  | |
| RVEDV index, mL/m² | 1.002 | 0.989 | 1.015 | | 0.742 | |
| RVESV index, mL/m² | 1.000 | 0.987 | 1.014 | | 0.968 | |
| RV ejection fraction, % | 0.996 | 0.971 | 1.021 | | 0.734 | |
| LVEDV index, mL/m^2^ | 1.006 | 0.992 | 1.020 | | 0.400 | |
| LVESV index, mL/m^2^ | 1.009 | 0.995 | 1.023 | | 0.208 | |
| LVEF, % | 0.985 | 0.946 | 1.026 | | 0.472 | |
| Positive mesocardial delayed-enhancement images | 1.675 | 0.668 | 4.202 | | 0.272 | |
| Positive transmural delayed-enhancement images | 1.068 | 0.410 | 2.784 | | 0.893 | |
| LV mass, g | 0.998 | 0.989 | 1.007 | | 0.683 | |
| LGE mass, g | 1.030 | 0.986 | 1.075 | | 0.183 | |
| ECV including positive delayed-enhancement images, % | 1.023 | 0.936 | 1.117 | | 0.621 | |
| ECV excluding positive delayed-enhancement images, % | 1.030 | 0.945 | 1.123 | | 0.499 | |
| iECV, mL/m^2^ | 1.021 | 0.981 | 1.064 | | 0.303 | |
| **Procedure data** |  |  |  | |  | |
| Cardiopulmonary bypass time, min | 1.008 | 0.995 | 1.022 | | 0.220 | |
| Cross-clamp time, min | 0.998 | 0.981 | 1.015 | | 0.808 | |

CI = confidence interval; HR = hazard ratio; other abbreviations as in Table 1, 2 and 3.
